# Praziquantel decreases fecundity in *Schistosoma mansoni* adult worms that survive treatment: evidence from a laboratory life-history trade-offs selection study

**DOI:** 10.1186/s40249-017-0324-0

**Published:** 2017-06-16

**Authors:** Poppy H.L. Lamberton, Christina L. Faust, Joanne P. Webster

**Affiliations:** 1 Institute for Biodiversity, Animal Health, and Comparative Medicine & Wellcome Centre for Molecular Parasitology, College of Medical, Veterinary & Life Sciences, University of Glasgow, G12 8QQ, Glasgow, UK; 2 London Centre for Neglected Tropical Disease Research, Department of Infectious Disease Epidemiology, School of Public Health, Imperial College London, St Mary’s Campus, W2 1PG, London, UK; 3 Centre for Endemic, Emerging and Exotic Diseases, The Royal Veterinary College, University of London, London, AL9 7TA UK

**Keywords:** *Schistosoma mansoni*, *Biomphalaria*, Mouse, Praziquantel, Resistance, Establishment, Survival, Fecundity, Trade-offs

## Abstract

**Background:**

Mass drug administration of praziquantel is the World Health Organization’s endorsed control strategy for schistosomiasis. A decade of annual treatments across sub-Saharan Africa has resulted in significant reductions of infection prevalence and intensity levels, although ‘hotspots’ remain. Repeated drug treatments place strong selective pressures on parasites, which may affect life-history traits that impact transmission dynamics. Understanding drug treatment responses and the evolution of such traits can help inform on how to minimise the risk of drug resistance developing, maximise sustainable control programme success, and improve diagnostic protocols.

**Methods:**

We performed a four-generation *Schistosoma mansoni* praziquantel selection experiment in mice and snails. We used three *S. mansoni* lines: a praziquantel-resistant isolate (R), a praziquantel-susceptible isolate (S), and a co-infected line (RS), under three treatment regimens: untreated, 25 mg/kg praziquantel, or 50 mg/kg praziquantel. Life-history traits, including parasite adult-worm establishment, survival, reproduction (fecundity), and associated morbidity, were recorded in mice across all four generations. Predictor variables were tested in a series of generalized linear mixed effects models to determine which factors had a significant influence on parasite life-history traits in definitive hosts under different selection regimes.

**Results:**

Praziquantel pressure significantly reduced adult-worm burdens across all generations and isolates, including within R-lines. However, previous drug treatment resulted in an increase in adult-worm establishment with increasing generation from P1 to F3. The highest worm numbers were in the co-infected RS line. Praziquantel treatment decreased adult-worm burden, but had a larger negative impact on the mean daily number of miracidia, a proxy for fecundity, across all three parasite isolates.

**Conclusions:**

Our predicted cost of resistance was not supported by the traits we measured within the murine host. We did not find evidence for negative adult worm density-dependent effects on fecundity. In contrast, of the adult worms that survived treatment, even low doses of praziquantel significantly reduced adult-worm fecundity. Such reductions in worm fecundity post treatment suggest that egg - based measures of drug efficacy, such as Kato-Katz, may overestimate the short-term effect of praziquantel on adult - worm burdens. These findings have important implications for *S. mansoni* transmission control, diagnostic protocols, and the potential for undetected selection toward drug resistance.

**Electronic supplementary material:**

The online version of this article (doi:10.1186/s40249-017-0324-0) contains supplementary material, which is available to authorized users.

## Multilingual abstracts

Please see Additional file 1 for translations of the abstract into the five official working languages of the United Nations.

## Background

Schistosomiasis is an infectious disease of poverty. The causative agents are platyhelminths of the class Trematoda. The majority of human intestinal schistosomiasis infections are caused by *Schistosoma mansoni*, a species found predominantly in Africa and South America. Eggs of *S. mansoni* are excreted in the stool. When faeces contaminate freshwater water through poor or lack of sanitation, the eggs hatch into miracidia. These miracidia infect *Biomphalaria* snail species where they reproduce asexually to produce cercariae. These cercariae are directly infective to humans; people are exposed when they contact infected water sources when bathing, swimming, fishing, and doing other water-based activities.

*Schistosoma mansoni* infection commonly causes anaemia, abdominal pain, and reduced physical and cognitive development. In up to 200,000 people per year, more severe chronic infections lead to complications such as hepatomegaly, splenomegaly, hepatic fibrosis, and/or cancer – leading to death in up to 20,000 people per year [1]. Morbidity from *Schistosoma* spp. infection is mainly caused by the density of eggs in the tissues, rather than adult worm numbers [2]. Schistosomiasis is second only to malaria in terms of its global parasitic morbidity and mortality burden and socioeconomic importance [3].

Over 230 million people are infected with schistosomiasis [4], with over 90% of these living in sub-Saharan Africa [5]. Efforts to improve water, sanitation, and hygiene facilities (WASH), as well as the control of snail populations, have been used to reduce transmission [6, 7]. However, the primary strategy currently endorsed by the World Health Organization (WHO) for lowering schistosomiasis burden and associated morbidity is Preventive Chemotherapy (PC) using praziquantel mass drug administration (MDA) [8, 9].

Low costs and large public-private partnership donations enable praziquantel’s use [10, 11] and over 52 countries have adopted this MDA strategy. In 2015, 66.5 million individuals worldwide received a MDA treatment for schistosomiasis, 81% of whom were school-aged children, and 87% were in Africa [12]. However, for *S. mansoni* the WHO endorsed 40 mg/kg praziquantel treatment has a parasitological cure rate of between 52 and 92% [13], with lower cure rates and egg reduction rates seen in areas which have received multiple annual treatments [14]. Under-dosing due to low drug absorption of the 40 mg/kg may also lead to even lower efficacy than previously thought, particularly in children due to pharmacokinetic factors [15, 16].

Annual MDA targeted at school-aged children has however been, in general, highly successful in reducing morbidity, prevalence and intensity in several regions, covering a range of baseline profiles [17–20]. This led the WHO to update their strategy in 2013 from morbidity control to elimination as a public-health problem [1, 9]. Nethertheless, some regions have reported little to no change in infection prevalence or intensity [14]. Monitoring the outcomes of MDA on intestinal schistosomiasis commonly involves parasitological examination of stool using Kato-Katz smears to confirm the presence of eggs, which are used as a proxy for adult worm numbers [21]. In areas where MDA has been successful, eggs are present in fewer individuals and/or the egg counts (representative of the intensity of infection) are lower. In areas where MDA has been less successful: egg counts can temporarily decline but there is little to no change in the number of people infected nor the long term mean infection intensities [14, 22]. The more sensitive point-of-care circulating cathodic antigen test (POC CCA) detects antigens from adult worms in active infections [23, 24]. POC CCAs show significantly lower cure rates than the Kato-Katz, and indicates that the positive linear association between adult worms and eggs might not hold post treatment [22]. This potentially indicates drug-induced embryostasis (the temporary or permanent cessation of egg production) as seen in onchocerciasis [25] and ascariasis [26].

Repeated drug treatments in the laboratory can lead to drug resistance developing [27, 28], with sporadic evidence of reduced treatment success in *S. mansoni* endemic communities [14, 29–32]. However, drug resistance is commonly associated with life-history costs such as reduced infectivity, survival and/or reproduction in helminths and other infectious agents [33–35]. Such costs are not only limited to drug resistance, but host-parasite trade-offs can occur throughout the life-cycle [36–41]. In areas where annual praziquantel treatment is ineffective at reducing the burden of schistosomiasis, it is important to pinpoint potential reasons underlying the failure of control strategies and these are likely to be affected by host-parasite-drug interactions and associated trade-offs.

We tested the prediction that *S. mansoni* exposed to multiple doses of praziquantel across multiple generations would display fitness costs, which could subsequently slow the spread or establishment of resistance in natural settings. We used a four generation *S. mansoni* selection study in laboratory mice and *Biomphalaria* snails to quantify the effects of in vivo praziquantel treatment on adult - worm establishment, survival, and fecundity. We compared praziquantel-susceptible and praziquantel-resistant parasite lines and the effects of varying levels of in vivo praziquantel exposure. We also predicted that with reduced intra-host competition post treatment due to lower worm numbers, we would see a relaxation of density dependence on adult - worm reproduction levels. The aim of the experimental selection study was to better understand the effect of long-term MDA, the associated risks of drug resistance developing and spreading, and other potential life-history effects of repeated mass treatments.

## Methods

### Details of host and parasites

#### Parasites

Two populations of *S. mansoni* isolates, originally obtained from infected people in Egypt in 1996 and subsequently undergoing multiple passages in the laboratory, were used to test the differences between susceptible and resistant parasite isolates. The putatively praziquantel-susceptible MOC isolate (henceforth S) was established from eggs excreted by a person living in the Nile region in Egypt prior to receiving a single 40 mg/kg praziquantel treatment that resulted in successful clearance of the parasite measured by Kato-Katz [42]. The parasite line has a mean ± standard deviation (SD) praziquantel ED_50_ of 80.0 ± 15 mg/kg in laboratory mice [43]. The second parasite isolate used in our experiment was EE2, an isolate established from eggs excreted by a person living in the Nile region in Egypt prior to three non-curative praziquantel treatments of 40 mg/kg, 40 mg/kg and then 60 mg/kg, respectively [42]. The resultant parasite line has a mean ± SD praziquantel ED_50_ of 212 ± 86 mg/kg in laboratory mice [43] and this isolate will be referred to as R for the remainder of the manuscript. The third parasite line was a mixed genotype infection with R and S (referred to as RS).

#### Definitive hosts

To reduce the influence of mice age, sex, and weight on the infectivity and development of cercariae, schistosomula, and adult worms [44], only female Tuc Ordinary (TO) Harlan® mice were infected, 7 days post arrival at the facility when they all weighed between 16 and 20 g. Mice were fed *ad libitum* on a uniform sterile diet of Clark’s Rat and Mouse food (CRM) (Lillico Ltd., UK). The mice paddled freely for 30 min in 100 ml of spring water containing the cercarial dose as described in detail in ‘Experimental design’ below.

#### Intermediate hosts

Laboratory snail lines of *Biomphalaria glabrata* and *B. alexandrina* were used to passage R, S, and RS. A laboratory strain of *B. glabrata*, (#2 strain) known to be highly susceptible to *S. mansoni* infection and laboratory mixed genotype *B. alexandrina* were used to reduce bottlenecking effects by increasing molluscan host heterogeneity [45, 46]. All snails were maintained at 24–25 °C and subjected to a light regime of 11 h light, 1 h dusk, 11 h dark and 1 h dawn [39, 47]. Individual snails were housed in individual plastic pots (10 cm × 8 cm × 5 cm) in 100 ml spring water (Iceland Ltd), changed weekly, and were provided with Styrofoam sheets where they preferentially deposit their eggs [48]. All snails were fed *ad libitum* on iceberg lettuce. All snails were maintained in isolation for a minimum of 1 week prior to miracidia exposure - this allowed acclimatization to individual conditions and differential auto- and allo-sperm storage [49, 50]. All snails were size-matched between treatment groups with a mean ± SD size of 10.19 ± 0.05 mm.

### Experimental design

**Fig. 1:**
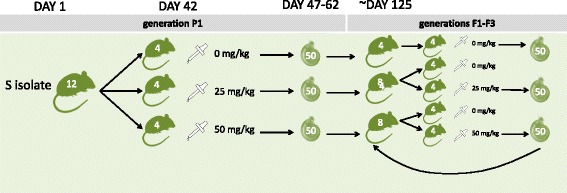
Experimental design for longitudinal selection of *Schistosoma mansoni* under in vivo praziquantel pressure (0, 25, or 50 mg/kg) for three parasite lines (susceptible (S), resistant (R), and a coinfected line (RS)). Praziquantel treatment was administered to the mice 42 days after parasite exposure. Mice were culled between days 47 and 62. Resultant miracidia were used to infect the snail at 6 miracidia/snail. In P1 mice were infected with 220 cercariae/mouse. For F1-F3, 110 cercariae/mouse were used. The experimental design shown here is an example showing only the parasite line started with the S isolate

Generation 1 (P1): Nine groups of four adult mice were exposed to 220 cercariae, a dose which had previously demonstrate high likelihood of infection with minimal unnecessary pathology in the mouse model, [39] of one of the three *S. mansoni* parasite lines: R, S, or RS (110 cercariae from each of S and R). These cercariae were pooled from snails 70 days post snail exposure to miracidia. Maturity of adult *S. mansoni* worms and subsequent egg production in mice takes approximately 42 days [51] and juvenile worms are not susceptible to praziquantel treatment. 42 days post-cercariae exposure, all mice were weighed, ear marked, and treated by oral gavage with 1) a sham 2% cremophor EL (control) dose, 2) a sub-curative low dose of 25 mg/kg praziquantel in 2% cremophor EL, or 3) a sub-curative medium dose of 50 mg/kg praziquantel in 2% cremophor EL (Fig. 1). Infected animals were euthanised 47 to 62 days post-cercariae exposure, using the schedule 1 method of cervical dislocation. Mice were euthanised prior to clinical morbidity, but post adult worm establishment, sexual maturity, and commencement of egg production. Adult schistosomes were recovered by a modified hepatic perfusion technique [52] and the worms were scored as paired or single, counted, sexed, and a subset’s length was measured (up to 10 of each of the paired, unpaired, male and female combinations). Weight of each mouse liver, spleen, and total body were recorded to the nearest mg.

The liver and spleen from each mouse were used to obtain eggs for hatching. These tissues of each mouse were macerated through a sieve in 250 ml 0.85% saline, left to sediment for 10 minutes, 200 ml supernatant was then removed and replaced with 200 ml saline. This was left to stand for a further 10 minutes and then the top 240 ml supernatant removed. The remaining sediment was washed out with 70 ml spring water and placed in direct light for 1 hour. Miracidia numbers, hatched from the eggs obtained from each mouse, were estimated in ten 0.2 ml samples per mouse.

Miracidia obtained from each of the four mice per experimental group were pooled and then used to infect 30 *B. glabrata* and 20 *B. alexandrina* snails per experimental line. Snails were individually exposed to six miracidia for 2 hours in 5 ml spring water. At 10 weeks post-miracidia exposure, the snails were kept in the dark for 24 h and then exposed to light to induce shedding of cercariae. Cercariae were pooled from ten snails per treatment group (including any *B. alexandrina* where possible).

Generation 2 (F1): 110 pooled cercariae from P1 snails were then used to infect four mice per experimental line. This dose was half that of the original P1 dose of 220 cercariae, due to unnecessary morbidity observed in the first generation. In addition to the original nine treatment groups, four extra mice served as untreated controls for each parasite line that had received praziquantel in the previous generation (Fig. 1). This enabled life-history traits of the parasite genotypes to be observed independently of a subsequent praziquantel dose, such as adult worm establishment and fecundity. These mice are termed Control2 mice. This protocol was continued until the fourth generation (F3) of mice.

Measurements recorded at culling are described above and include: (i) weight of mouse body, liver and spleen; (ii) virulence proxy (liver and spleen as a proportion of total weight) [53]; (iii) number of worms with details on paired status, sex and length (subset); and (iv) mean number of miracidia obtained from the liver and spleen of each mouse 1 hour after hatching (proxy for fecundity).

### Statistical analyses

Parasite life-history traits were modelled as outcome variables using generalized linear mixed effects models (GLMMs). The focus for the analysis was on adult establishment (number of adult worms), average daily worm pair miracidial output (hatched eggs), and host virulence (proportion of mouse weight that is spleen and liver). Adult establishment and miracidial output were modelled with the *nbinom1* family and *logit* link function using the ‘glmmadmb’ function in the package *glmmADMB* (http://glmmadmb.r-forge.r-project.org/). Virulence data were log transformed and analysed using a Gaussian distribution with *lmer* in *lme4* package [54]. In all models, treatment group was included as a random effect. Predictor variables included: (i) experimental treatments (parasite line, praziquantel dose, praziquantel selection line (praziquantel dose in current or previous generation), laboratory generation); (ii) definitive host variables (weight at treatment, weight at cull, percentage change in mouse weight, number of days post treatment for culling); and (iii) parasite characteristics (length of worms (paired, unpaired, male, female)). Model selection was performed in a stepwise manner, using Akaike information criterion (AIC) to compare between models of different complexities. Final models were checked for overdispersion. For models that used a negative binomial distribution, estimates were converted to incidence rate ratios (IRR) to improve interpretation and 95% confidence intervals were calculated.

## Results

### Worm establishment and survival

Adult worm numbers were measured between days 47–62 post infection, equating to 5–20 days post praziquantel treatment (mean = 9.9 days). Although cull day post treatment affected the establishment and survival of adult worms in a univariate model (IRR: 0.96; 95% *CI*: 0.94, 0.99), it was not significant once included in multivariate models or present as a predictor in the best fit model. Because different cercarial doses were used between P1 (220 cercariae/mouse) and F1-F3 (110 cercariae/mouse), the number of adult worms was standardized to compare between generations. The total number of worms measured integrates both the establishment and survival of adult worms to the cull date and hence we were unable to distinguish between these two processes in this analysis of the mice.

The best-fit model to explain standardized adult worm abundance included parasite line, praziquantel selection in current or previous generation, the percentage weight change between treatment and culling, and generation (Table 1). Adult worm numbers were highest in the RS line (IRR: 1.33, 95% *CI*: 1.15, 1.53) compared to S line, but there was no significant difference between R and S lines (IRR:1.05, 95% *CI*:0.91, 1.23). Praziquantel selection from treatment in the current generation, successfully decreased adult worm numbers in both the low (IRR: 0.83, 95% *CI*: 0.71, 0.97) and medium (IRR: 0.80; 95% *CI*: 0.68, 0.94) praziquantel dose groups. Greater weight loss after treatment was associated with a reduction in worm abundance (IRR: 0.17; 95% *CI*: 0.083, 0.33). However, in each generation, overall worm establishment and/or survival (being unable to differentiate these) increased (IRR: 1.26, 95% *CI*: 1.19,1.34).

**Table 1:** The estimates for each of the predictor variables included in the best-fit multivariate generalized linear mixed models (GLMMs) for the standardized total number of adult *Schistosoma mansoni* adult worms and daily miracidia per worm pair

Variable	*Number of adult worms*	*Daily hatched miracidia* per pair
	IRR (95% *CI*)	IRR (95% *CI*)
Intercept	14.56***	21.53***
	(11.11, 19.08)	(18.20, 25.47)
Parasite line (baseline: S)		
R	1.06 (0.91, 1.23)	0.95 (0.76, 1.18)
RS	1.33*** (1.15, 1.53)	1.21 (0.98, 1.50)
Praziquantel selection (baseline: control)		
Low selection	0.83* (0.71, 0.97)	–
High selection	0.80** (0.68, 0.94)	–
Praziquantel treatment (baseline: 0 mg/kg)		
25 mg/kg	–	0.37*** (0.29, 0.47)
50 mg/kg	–	0.26*** (0.20, 0.33)
Generation	1.26*** (1.19, 1.34)	–
Proportion weight change	0.17*** (0.08, 0.33)	–

*IRR* incidence rate ratio, *CI* confidence interval, * *P* < 0.05, **P < 0.01, ***P < 0.001

### Adult worm sex ratios and sizes

**Fig. 2:**
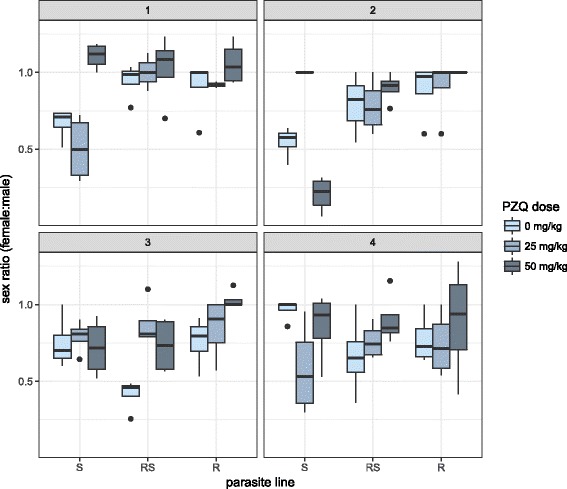
Sex ratios of *Schistosoma mansoni* adult worms across four generations for each parasite line and praziquantel treatment regime. Each panel of the figure represents one of the four generations labelled from 1 (P1) through to 4 (F3). Values above 1 indicate an excess of females, whilst values below 1 indicate an excess of males

Throughout the experiment, unpaired females were rarely found. The sex ratio (number of females: number of males) was often below 1, meaning an excess of unpaired males. For parasite lines that were exposed to the consistent praziquantel selection pressure throughout all four generations (Fig. 2, Control2s are not shown), there was an increase in the female: male ratio with increasing generation, and within parasite lines as the praziquantel dose increased. In P1, more female worms survived the 50 mg/kg treatment than males in all three parasite lines, with the greatest difference in S. This trend was not seen again in any of the three subsequent treated generations. We also measured the length of adult worms to determine if the size of worms affected their ability to persist and produce eggs after praziquantel treatment. There were no significant differences between parasite lines or praziquantel treatment groups in worm size (unpaired males, paired males, unpaired females, and paired females or overall) and worm size was not a significant predictor of worm establishment or fecundity (Additional file 2: Figure S1).

### Daily worm fecundity

**Fig. 3:**
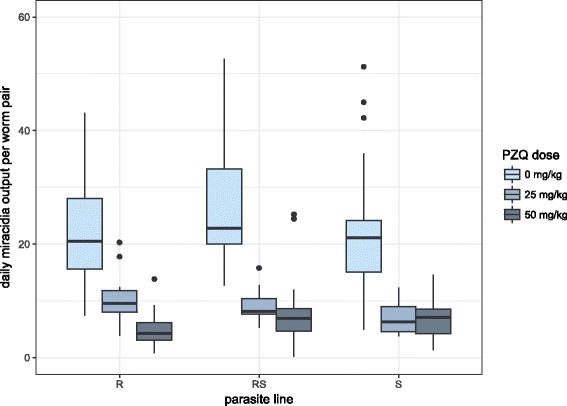
Daily mean viable *Schistosoma mansoni* miracidia output per worm pair (fecundity). Each boxplot summarizes the daily miracidial output per pair across four generations for each parasite line and praziquantel treatment dose

Fecundity of worm pairs was measured by hatching eggs recovered from the spleen and liver of mice. As samples were not all collected on the same day across all experimental groups, in order to standardize rates, total miracidia estimates were divided by days post-treatment and the number of worm pairs. The best-fit model for fecundity included praziquantel dose and parasite isolate (Table 1). Treatment with praziquantel was associated with a significantly lower fecundity (low praziquantel IRR: 0.37, medium praziquantel IRR: 0.24) compared with control mice (Fig. 3). There was no significant difference between parasite lines: RS had a non-significant slightly higher (IRR = 1.21, 95% *CI*: 0.98, 1.50) fecundity compared to the S line, whereas R was not different (IRR = 0.95, 95% *CI*: 0.76, 1.18) from S. Although differences in parasite line effects were not significant individually, including parasite line in the model significantly improved the fit. We did not find support for density-dependent effects on fecundity: viable miracidia production per worm was not significantly linked to either worm pair numbers or total worm burden (Additional file 2: Figure S2).

### Morbidity of definitive hosts

Virulence of parasites in definitive hosts was estimated using the proportion of a mouse’s body weight that was liver and spleen. This standard metric reflects not only the immunogenic eggs released by adult worms, but also inflammation and immunopathology induced during infection [2]. Virulence in a mouse significantly increased with the logarithm of the number of miracidia recorded (estimate = 1.22, SE = 0.42, Table 2). Praziquantel treatment was positively associated with higher virulence, but the lower praziquantel dose had a larger effect (2.11, SE = 0.43) than the higher dose (1.54, SE = 0.46). As the relative size of the liver and spleen was used as a virulence proxy and measurements were taken before eggs were removed, we checked to assess if this was a confounding variable. However, total miracidia was not related to virulence in a predictable manner (Additional file 2: Figure S3). Although alternative models were fit without total miracidia, none were as parsimonious as the model only including praziquantel dose and miracidia numbers.

**Table 2:** The estimates for predictor variables from the best fit linear mixed model for virulence associated with *Schistosoma mansoni* infection in mice

Predictor Variables	*Virulence* percentage of weight that is liver and spleen
	Est (SE)
Intercept	4.7** (1.43)
Praziquantel treatment (baseline: 0 mg/kg)	
25 mg/kg	2.11*** (0.43)
50 mg/kg	1.54** (0.46)
log_10_ (total miracidia)	1.22** (0.42)

*SE* standard error; * *P* < 0.05, **P < 0.01, ***P < 0.001

## Discussion

We characterized, within a laboratory experimental setting, adult - worm establishment, fecundity, and virulence of praziquantel-susceptible and praziquantel-resistant *S. mansoni* lines in their (murine) definitive hosts, under in vivo praziquantel pressure and in untreated groups. In vivo praziquantel treatment significantly reduced fecundity in surviving adult worms, but with no significant difference between parasite lines. Daily fecundity per worm pair was negatively associated with in vivo praziquantel concentration. This reduction in fecundity with treatment has important implications for control programs and the monitoring of *S. mansoni* drug efficacy using egg based diagnostics alone.

In endemic communities, parasitological examination of the stool, most commonly the Kato-Katz thick smear technique [21], is used to evaluate praziquantel efficacy [55]. Improved statistical analyses help to accurately characterise egg reduction rates [56, 57] and the effects of multiple MDA rounds [14]. However, Kato-Katzs have poor sensitivity at low infection intensities and post treatment [22, 58, 59]. If adult worms survive treatment, but have a lower fecundity, then Kato-Katzs and other egg based diagnostic methods may further indicate a greater reduction in worm burden (infection intensity, measured as egg per gram of stool are taken as a proxy for adult worm numbers) than has actually occurred.

Our laboratory findings here strongly support field based research where, for example, adult worm antigen diagnostics (POC CCA) demonstrated significantly lower cure rates than Kato-Katzs, which were unlikely to be explained by diagnostic sensitivity alone [22]. From our field results we could not differentiate between adult worms surviving treatment but with a cessation of egg production (embryostasis) from juvenile worm infections that had not yet become egg patent [22]. If praziquantel induces a degree of embryostasis, and this is permanent, or semi-permanent, then from a virulence standpoint this will result in lower egg numbers and therefore lower associated morbidity and transmission [2] even though the mechanism involved will be different to that of worm death. However, if such embryostasis is only temporary, then it could have far greater implications, and be a form of drug resistance that would contribute to the future genepool whilst being undetected by standard egg diagnostic methods. This in turn may even result in fecundity compensation when egg production is reinstated, that could be detected by genetic analyses of offspring, where intensities are similar to pre-treatment levels, but from a smaller effective breeding population (Gower et al., in preparation). This highlights the importance of studies such as ours in understanding the complex effects of drug treatment and selection.

Our field and laboratory studies to date cannot confirm if this potential embryostasis is permanent or temporary, but genetic studies of the miracidia offspring using sibship analyses to infer parental genotypes could inform if future eggs are from new or surviving worms. Such a drug induced reduction in fecundity, without death or damage to the adult worms, has been reported for schistosomes using nicarbazin [60], but not previously reported for praziquantel. Such potential embryostasis is further supported by a link between an agent (United States Patent 6,514,963) that inhibits egg production in schistosomes and also inhibits the influx of calcium through cell membrane channels, which are thought to be associated with the action of praziquantel [61].

One limitation of our study is that the effect of praziquantel directly on schistosome eggs is not completely understood, and what we report as a reduction in fecundity, measured here as daily miracidia per worm pair, could be an artefact of eggs becoming non-viable with praziquantel treatment [62] and we could be underestimating the daily egg production per worm pair. In contrast, if egg production had begun several days before we treated with praziquantel, then we would be overestimating fecundity.

In addition to the fecundity reductions discussed above, praziquantel treatment also reduced adult - worm survival, in particular lowering the number of male worms seen as an increase in the female to male worm ratio (Fig. 2). This finding supports studies reporting higher survival in females following praziquantel treatment [63, 64], potentially due to the location of females, being physically protected by males in their gynecophoric canal and therefore less susceptible to praziquantel [65]. This was particularly apparent in the first generation (P1), where S had a rare surplus of females after the 50 mg/kg praziquantel treatment dose. Changes in sex ratio with treatment could also impact host morbidity, reproductive success of the parasite and could have implications for future adaptation. For example, with an imbalanced sex ratio, mate swapping [66] could increase parasite heterogeneity without increasing overall egg numbers, particularly with males capable of mating with multiple females [67]. Such mate swapping cannot be detected in humans by standard parasitological techniques but could be detected by population genetic approaches. Such increased outbreeding in schistosomes has been previously demonstrated through resistance selected snails in the laboratory [40]. Although sex sensitivity can also vary by strain [62] we did not observe any significant differences between parasite lines, potentially due to the sub-curative doses we were administrating, which were imposed to increase selective pressure for potential drug resistance.

Previous laboratory studies have shown both the loss of resistance in the absence of praziquantel exposure [68] and the gain of resistance phenotypes in susceptible lines under selection in as few as six laboratory generations [27]. In our selection study parasite line was a better predictor of selection, in this instance worm establishment, than in vivo praziquantel dose. For example, the S line that had been exposed to high praziquantel doses in previous generations, but not in the current generation, had indistinguishable adult - worm establishment and survival from those which continued to receive praziquantel treatment.

Although R had higher adult survival compared with S, there was not a significant difference between fecundity and virulence (when unexposed to praziquantel). RS, on the other hand, had significantly higher adult worm numbers and higher fecundity. This may be due to outbreeding of two potentially inbred laboratory lines, rather than innate fitness benefits. The S and R isolates had been maintained in the laboratory for many generations and are likely to have undergone a population bottleneck [69]. We also observed higher adult - worm establishment per cercaria with increasing generation. A potential explanation for this is that the initial worm exposure burdens were higher due to larger cercarial exposure (220 cercariae vs. 110 cercariae in subsequent generations) and subsequent density - dependent effects may have limited adult establishment in P1.

Morbidity from *Schistosoma* infections is mainly caused by the density of eggs in the tissues, rather than the presence of the adult worms [2]. In this study, virulence was best predicted by dose of praziquantel and total egg burden. In humans, the severity of schistosomiasis symptoms is related to infection intensity [70], host immune response [71] and parasite genotype [72]. Here we observed no significant difference between morbidity indicators and parasite line, eluding to the fact that if drug resistance develops, associated morbidity should not worsen.

Here we show that a reduction in fecundity, most significantly affected by praziquantel treatment, has positive benefits for murine hosts in the short term. In contrast, praziquantel treatments increased our virulence estimates. As praziquantel works synergistically with the immune system [73–75] and the spleen is associated with the production of white blood cells, then a possible explanation for the increase in spleen weight with treatment could be the effect of praziquantel acting in conjunction with the immune system. This is further supported by field observations in Burundi, where praziquantel treatment successfully reduced periportal fibrosis and hepatomegaly but was associated with an increase in splenomegaly up to 2 years after treatment with praziquantel [76]. Also, in Sudan the percentage of patients with hepatomegaly decreased significantly up to 2 years after praziquantel treatment but splenomegaly remained unchanged [77]. Correlations between *S. mansoni* infection intensity and morbidity are not clearly delineated and may be altered with chemotherapy. Our study highlights the complexity of co-examining morbidity and changes in infectious status and intensities, and supports potential independent evaluation of such measures in community based control programmes [78].

Previous studies have demonstrated weak costs of schistosome resistance to praziquantel in the definitive mouse host, or indeed benefits, to be often mirrored by greater costs in the molluscan host [68]. We did not observe life-history costs associated with praziquantel resistance in murine hosts. Indeed, traits associated with R were higher worm numbers in the definitive mouse host, however this may be intrinsically linked to greater negative trade-off traits in intermediate snail hosts [79, 80]. Transmission of schistosomiasis is reliant on the fitness of the parasite in both the definitive and intermediate hosts. Schistosomes can be highly virulent to their snail hosts, raising mortality rates and reducing host reproduction by exploiting reproductive tissues [36, 37, 39, 79, 81, 82]. Investigations into the molluscan life-cycle stages of these parasite lines as selection occurs will help evaluate whether the praziquantel selective pressures are propagated through the next stage of the life cycle.

Our study used *S. mansoni* parasite lines and although it is likely that other schistosome species may behave similarly, this cannot be substantiated without further research. Additional studies using a more diverse range of susceptible and resistance parasite lines (*S. mansoni* and other spp.) would help to support any differences observed here.

## Conclusion

This study has highlighted a significant decrease in fecundity in both praziquantel-susceptible and praziquantel-resistant *S. mansoni* lines with treatment. The effect of praziquantel had a larger impact on fecundity than adult worm survival. This has important public health implications for monitoring drug efficacy in control programs, as standard egg based diagnostics, such as Kato-Katz may over estimate drug efficacy in comparison to adult worm antigen detecting methods, such as POC CCA. Our findings also demonstrated that drug resistance was not associated with significant life-history costs in the murine host. This could indicate that if drug resistance is selected for in the field it may be more likely to spread, potentially without being detected. However further work on potential costs in the snail hosts are warranted, as well as genetic studies on parasites being excreted post treatment to establish if *S. mansoni* embryostasis occurs in treated humans, and if so, if the worms go on to contribute these potential resistant parasites into the gene pool or if egg cessation is permanent.

## Additional files

Additional file 1: Lamberton et al. 2017 IDoP Multilingual abstracts in the five official working languages of the United Nations. (PDF 861 kb)

Additional file 2: Lamberton et al. 2017 IDoP Supplementary Figures 1, 2 and 3. (DOCX 322 kb)

Additional file 3: Lamberton et al. 2017 IDoP Raw mouse and worm data from experimental infections. (XLSX 242 KB)

## Abbreviations

AIC: Akaike information criterion
CI: Confidence interval
GLMM: Generalized linear mixed models
IRR: Incidence rate ratio
MDA: Mass drug administration
PC: Preventative Chemotherapy
POC CCA: Point-of-care circulating cathodic antigen test
SD: Standard deviation
WHO﻿: World Health Organization
WASH﻿: Water, Sanitation, and Hygiene
