# Supplementary material for: Praziquantel decreases fecundity in Schistosoma mansoni adult worms that survive treatment: evidence from a laboratory life-history trade-offs selection study
Source: Infect Dis Poverty. 2017 Jun 16;6:110. doi: 10.1186/s40249-017-0324-0 (PMC5472905; doi:10.1186/s40249-017-0324-0)
Supplement: Supplementary file 1 — Lamberton et al. 2017 IDoP Multilingual abstracts in the five official working languages of the United Nations. (PDF 861 kb) [file 40249_2017_324_MOESM1_ESM.pdf]

Translation of the abstract into the five official working languages of the United Nations

برازيكوانتيل يقلل الخصوبة في ديدان البلهارسيا المعوية البالغة التي ظلت على قيد الحياة بعد العلاج: دليل من دراسة مختبرية لتاريخ الحياة لاختيار العلاج

الـ عربـ ي الـمـلخص

خلفية: ان اعطاء العلاج الجماعي من البرازيكوانتيل هي استراتيجية منظمة الصحة العالمية المعتمدة لمكافحة البلهارسيا. وقد أدت العلاجات السنوية خلال عقد من الزمان في جنوب الصحراء الكبرى في أفريقيا إلى انخفاض كبير في معدلات انتشار العدوى وكثافتها، على الرغم من أن "المناطق الساخنة" لا تزال موجودة. و قد يحدث تكرار اعطاء العلاج الى ضغوطا انتقائية قوية على الطفيليات، مما قد يؤثر على سمات تاريخ الحياة التي تؤثر على اليات انتقال العدوى. أن فهم الاستجابة للعلاج وتطور سمات هذه الاستجابة يمكن ان يساعد في تقديم المعلومات بشأن كيفية تقليل خطر مقاومة الأدوية إلى أدنى حد ممكن، وتحقيق أقصى قدر من النجاح في برنامج المراقبة المستدامة، وتحسين بروتوكولات التشخيص.

الطريقة: تم اجراء التجربة على أربعة اجيال من البلهارسيا المعوية لاختيار البرازيكوانتيل في الفئران والقواقع. و قد تم استخدام ثلاث عزلات من البلهارسيا المعوية: مقاومة للبرازيكوانتيل(ر) , قابلة للعلاج بالبرازيكونتيل (س) وخط مصاب بالعدوى (رس) تحت ثلاثة نظم علاجية: غير معالجة, معالجة ب 25 ملغم / كغم برازيكوانتيل، أو 50 ملغم / كغم برازيكوانتل
، ،. وسجلت الفئران في جميع الأجيال الأربعة سمات تاريخ الحياة، بما في ذلك وجود الدودة البالغة ، وبقائها، ومعدلات التكاثر (الخصوبة)، و ما تسببه من حالة مرضية مرتبطة بها. تم اختبار متغيرات التنبؤ في سلسلة من نماذج التأثيرات الخطية لتحديد العوامل التي لها تأثير كبير على طفيليات سمات الحياة في العائل النهائي تحت أنظمة اختيار مختلفة

الاجيال. ومع ذلك، أدى فقد ادى تكرار العلاج **النتائج**: أدى استعمال البرازيكوانتيل بكثرة الى انخفاض كبير في أعداد الديدان البالغة عبر جميع السابق إلى زيادة في اعداد الديدان البالغة من الجيل الأول حتى الجيل الثالث.
وكانت أعلى أعداد للديدان في خط (رس) المصاب بالعدوى. وقد أدى علاج برازيكوانتيل إلى انخفاض عبء الديدان البالغة، ولكنه كان له تأثير سلبي أكبر على متوسط العدد اليومي للميراسيديا، وهو بديل للخصوبة، في جميع عزلات الطفيليات الثلاث.

الاستنتاجات: لم تكن التكلفة المتوقعة للمقاومة مدعومة بالصفات التي قمنا بقياسها داخل الفئران. لم نجد أدلة على الآثار السلبية على الخصوبة و التي تعتمد على كثافة دودة البالغين. في المقابل، من الديدان البالغة التي نجت من العلاج، الجرعات المنخفضة من البرازيكوانتيلقد ادت الى انخفاض كبير في الخصوبة. هذا الانخفاض في الخصوبة بعد العلاج يشير إلى أن قياس فعالية الدواء،بواسطة الاختبارات التي تعتمد على عدد البيض مثل اختبار كاتو كاتس، قد بالغ في تقدير تأثيرالبرازيكوانتيل على المدى القصير على عدد الديدان البالغة. هذه النتائج لها آثار هامة في السيطرة على انتقال البلهارسيا المعوية وايضا على البروتوكولات التشخيصية وإمكانية الاختيار غير المعلن نحو مقاومة العقار.

Poppy H.L. Lamberton, Christina L. Faust, Joanne P. Webster

吡喹酮可降低治疗后存活的曼氏血吸虫成虫的繁殖力：来自实验室生活史权衡选择研究的证据

Poppy H.L. Lamberton, Christina L. Faust, Joanne P. Webster

摘要

引言：使用吡喹酮进行群体治疗(mass drug administration, MDA)是世界卫生组织认可的血吸虫病防控策略。尽管“高发区”仍然存在，在撒哈拉以南非洲地区的持续十年治疗已大大降低了血吸虫感染率和感染度。重复的药物治疗对寄生虫施加了巨大的选择性压力，可能会影响寄生虫的生活史特征进而影响传播动力学。了解药物治疗反应和这些特征的演变有助于了解如何将抗药性发展风险降到最低，最大限度地实现可持续防治计划的成功，并改进诊断方案。

方法：我们在小鼠和钉螺中进行了 4 轮曼氏血吸虫的吡喹酮选择实验。使用 3 种曼氏血吸虫分离株，即吡喹酮抗性株（R），吡喹酮敏感株（S）和共感染株（RS）执行 3 种治疗方案：未治疗、给予 25 mg/kg 吡喹酮或 50 mg/kg 吡喹酮进行治疗。在 4 代小鼠中记录了生活史特征，包括分离株的建立、生存、繁殖（繁殖力）和相关的发病率。在一系列广义线性混合效应模型中测试预测因子变量，以确定哪些因素在不同选择方式下的寄生虫生活史有重要影响。

结果：吡喹酮的选择压力显著降低了所有代和分离株的成虫虫荷，包括 R 分离株。然而，前期药物治疗导致各分离株的建立，从 P1 代繁殖到 F3 代。共感染的 RS 虫株的成虫数量最多。吡喹酮治疗降低了成虫虫荷，但对这 3 种寄生虫分离株平均每天产生毛蚴数量（繁殖力的代表）造成了较大的负面影响。

结论：在小鼠宿主所得到的生活史特征无法支持我们预测的抗药性成本。我们也没有找到证据来证明成虫密度依赖对繁殖力的负面影响。相反，在治疗后存活下来的成虫中，即使低剂量的吡喹酮也显著降低了成虫的繁殖力。这种治疗后降低成虫繁殖能力的结果提示，基于虫卵的药效检测方法，

如 Kato-Katz 法，可能会高估吡喹酮对成虫虫荷的短期影响。这些发现对曼氏血吸虫传播控制、诊断方案以及未被发现的药物抗性选择具有重要意义。

Translated from English version into Chinese by Hua Liu, edited by Pin Yang

Le praziquantel diminue la fécondité chez les vers adultes de *Schistosoma mansoni* qui survivent au traitement: évidences d'une étude de sélection des compromis des traits d'histoire de vie en laboratoire

Poppy H.L. Lamberton, Christina L. Faust, Joanne P. Webster

Résumé
Contexte: Le traitement de masse au moyen de praziquantel est la stratégie de contrôle approuvée par l'Organisation Mondiale de la Santé pour la schistosomiase. Une décennie de traitements annuels en Afrique sub-Saharienne a entraîné des réductions importantes de la prévalence et des niveaux d'intensité d'infection, mais des ‘point chauds/hot-spots’ restent. Les traitements médicamenteux répétés posent de fortes pressions sélectives sur les parasites, ce qui peut affecter les traits d'histoire de vie qui ont une incidence sur la dynamique de transmission. La compréhension des réponses développées suite à la prise du médicament et l’évolution de ces traits induite par le médicament peuvent renseigner sur comment minimiser les risques de développement de résistance au médicament, maximiser la réussite d’un programme de gestion durable et améliorer les protocoles de diagnostic.
Méthodes: Une expérience de sélection au praziquantel a été réalisée sur quatre générations de *Schistosoma mansoni* chez des souris de laboratoire et des escargots. Nous avons utilisé trois isolats de *S. mansoni*: résistant au praziquantel (R), sensible au praziquantel (S) et une ligne co-infectée (RS), sous trois schémas de traitement: non traité, 25 mg/kg praziquantel ou 50 mg/kg praziquantel. Les traits d'histoire de vie, y compris l'établissement de parasites adultes, la survie, la reproduction (fécondité) et la morbidité associée, ont été mesurés chez la souris au cours des quatre générations. Les variables prédictives ont été testées dans une série de modèles linéaires généralisés à effets mixtes pour déterminer quels facteurs ont une influence significative sur les traits d'antécédents parasitaires dans les hôtes définitifs selon différents régimes de sélection.
Résultats: La pression praziquantel a considérablement réduit les charges de vers adultes pour toutes les générations et les isolats, y compris les lignées R. Cependant, l’exposition préalable au médicament praziquantel induit une augmentation de l'établissement des vers adultes de la génération P1 à F3. Les nombres de vers les plus élevés se trouvaient dans la lignée RS co-infectée. Le traitement par praziquantel a diminué la charge/l’intensité des vers adultes, mais a eu un impact négatif plus important sur le nombre quotidien moyen de miracidia, un proxy de la fécondité, pour les trois isolats de parasites.
Conclusions: Notre prédiction du coût de la résistance n'a pas été observée pour les traits que nous avons mesurés dans l'hôte murin. Nous n'avons pas trouvé d’évidence pour les effets négatifs de la densité de vers adultes sur la fécondité. À l'opposé, les vers adultes qui ont survécu le traitement, même à faibles doses de praziquantel, avaient une fécondité significativement réduite. De telles réductions dans la fécondité des vers après traitement suggèrent que les mesures de l'efficacité du médicament basées sur les œufs, telles que le Kato-Katz, peuvent surestimer l'effet à court terme du praziquantel sur les charges de parasites adultes. Ces résultats ont des implications importantes pour le contrôle de la transmission de *S. mansoni*, les protocoles de diagnostic et le risque d’absence de détection d’une sélection vers une résistance au médicament.

Празиквантел уменьшает плодовитость взрослых червей *Schistosoma mansoni*, которые выживают при лечении: результаты лабораторного эксперимента по отбору.

Poppy H.L. Lamberton, Christina L. Faust, Joanne P. Webster

Абстракт

Введение: Массовое применение препарата празиквантел является одобренной Всемирной организацией здравоохранения стратегией борьбы с шистосомозом. Десятилетие ежегодных применений в странах Африки к югу от Сахары привело к значительному снижению уровня распространенности инфекции и ее интенсивности, хотя до сих пор остаются «горячие точки». Повторные медикаментозное лечение оказывают сильное селективное давление на паразитов, что может повлиять на их особенности, влияющие на динамику передачи болезни. Понимание динамики ответов на лекарственные средства и эволюция отдельных признаков признаков могут помочь в свести к минимуму риск развития лекарственной устойчивости, максимизировать устойчивость успеха программы контроля и улучшить диагностику.
Методы: Мы провели эксперимент длиной в 4 поколения по отбору *Schizosoma mansoni* по реакции на празиквантел используя мышей и улиток. Мы использовали три изолята *S. mansoni*: устойчивый к празиквантелу (R), празиквантел-восприимчивый (S) и ко-инфицированную линию (RS), при трех режимах лечения: необработанный, 25 мг / кг празиквантела или 50 мг / кг празиквантела. Такие показатели как численность, выживание, плодовитость регистрировались на в мышиной модели на протяжении 4 поколений. Предикторы эффектов были протестированы в серии обобщенных моделей линейных смешанных эффектов, чтобы определить, какие факторы оказали значительное влияние на характеристики жизненного цикла паразита у конечных хозяев при разных режимах отбора.
Результаты: применение празиквантела значительно уменьшило паразитическую нагрузку взрослых червей во всех поколениях и изолятах в том числе внутри R-линий. Однако, предыдущее лечение приводило к увеличению числа взрослых червей с увеличением генерации от P1 до F3. Наибольшее число червей находилось в коинфицированной линии RS. Лечение празиквантелом уменьшило численность взрослых червей, но оказало большее отрицательное влияние на среднесуточное количество личинок, прокси-фактора для плодовитости, во всех трех изолятах паразитов.
Выводы: Прогнозируемая цена лекарственной устойчивости не наблюдалась в совокупности признаков, которые мы измеряли в экспериментах на мышах. Мы не нашли доказательств негативного плотностно- зависимого влияния на плодовитость у червей. Напротив, у взрослых червей, выживших при лечении, даже низкие дозы празиквантела значительно уменьшали плодовитость. Такие результаты лечения предполагают, что меры, основанные на подсчете яиц для оценки эффективности препарата, как например, Като-Кац, могут переоценить краткосрочный эффект применения празиквантела на численность червей у взрослых. Эти результаты имеют важное значение для контроля передачи *S. mansoni*, протоколов диагностики и возможности детекции незаметного отбора к лекарственной устойчивости.

El praziquantel disminuye la fecundidad en los gusanos adultos de *Schistosoma mansoni* que sobreviven al tratamiento: evidencia de un estudio de selección de compensaciones de vida biológica de laboratorio

Poppy H.L. Lamberton, Christina L. Faust, Joanne P. Webster

Resumen

Antecedentes: La administración masiva del fármaco praziquantel es la estrategia aprobada por la Organización Mundial de la Salud para el control de esquistosomiasis. Una década de tratamientos anuales en el África subsahariana ha resultado en reducciones significativas de la prevalencia y niveles de intensidad de infección, a pesar de la persistencia de ‘hotspots’. Tratamientos repetidos con fármacos ejercen fuertes presiones selectivas sobre parasitos, las que pueden afectar rasgos de historia de vida que impactan las dinámicas de transmisión. La comprensión de las respuestas a tratamientos farmacológicos y la evolución de dichas características, puede ayudar a entender como minimizar el riesgo de resistencia a los medicamentos, maximizar el éxito de programas de control y mejorar protocolos de diagnosis.
Métodos: Llevamos a cabo un experimento de selección de *Schistosoma mansoni*/praziquantel durante cuatro generaciones en ratones y caracoles. Usamos 3 linajes de *S. mansoni*: resistente a praziquantel (R), susceptible a praziquantel (S), y linaje coinfectado (RS); bajo tres regímenes de tratamiento: sin tratar, 25 mg praziquantel/kg, o 50 mg praziquantel/kg. A travez de cuatro generaciones de ratones registramos rasgos de historia de vida, incluyendo el establecimiento de parásitos adultos, supervivencia, reproducción (fecundidad), y morbilidad asociada. Variables independientes fueron puestas a prueba en una serie de

modelos lineares generalizados mixtos para determinar que factores tuvieron una influencia significativa en los rasgos de historia de vida de los hospedadores definitivos bajos diferentes regímenes de selección.
Resultados: La presión selectiva de praziquantel redujo significativamente las cargas de gusanos adultos en todas las generaciones y linajes, inclusive en los linajes R. Sin embargo, tratamiento farmacológico previo resultó en un aumento en el establecimiento de gusanos adultos a medida que las generaciones incrementaban, de P1 a F3. Las abundancias de parásitos más altas ocurrieron en el linaje RS. El tratamiento con praziquantel redujo la carga de gusanos adultos, pero tuvo un impacto negativo mayor en el promedio diario de miracidia, un indicador de fecundidad, en los tres linajes de parásitos.
Conclusiones: Nuestra predicción de costo de la resistencia no fue apoyada por los rasgos medidos en en el hospedador murino. No encontramos evidencia de la densidad del parásito adulto sobre la fecundidad. En contraste, incluso bajas dosis de praziquantel redujeron significativamente la fecundidad en aquellos gusanos adultos que sobrevivieron el tratamiento. Dichas reducciones en fecundidad post tratamiento sugieren que medidas de eficiencia de fármacos basadas en huevos, como Kato-Katz, podrían sobreestimar el efecto a corto plazo de praziquantel en la abundancia de parásitos adultos. Estos hallazgos acarrean implicaciones importantes para el control de la transmisión y protocolos de diagnóstico de *S. mansoni*, así como también en la potencial selección no detectada para resistencia de fármacos.

Translated from English version into Spanish by Sebastián Muñoz
