# Supplementary material for: Praziquantel decreases fecundity in Schistosoma mansoni adult worms that survive treatment: evidence from a laboratory life-history trade-offs selection study
Source: Infect Dis Poverty. 2017 Jun 16;6:110. doi: 10.1186/s40249-017-0324-0 (PMC5472905; doi:10.1186/s40249-017-0324-0)

**Supplementary Information**

**Figure S1. Average size of *Schistosoma mansoni* adult worms across all four generations and all nine treatment groups.** The data presented include length measurements of up to 10 worms from each category combination (paired/unpaired, male/female). A single mouse could have up to 40 individual worms measured. There was a large amount of variation in the size of worms observed – both between treatment groups and across generations. However, there are no significant differences between groups, even when considering only paired individuals, only single individuals, only males or only females.**
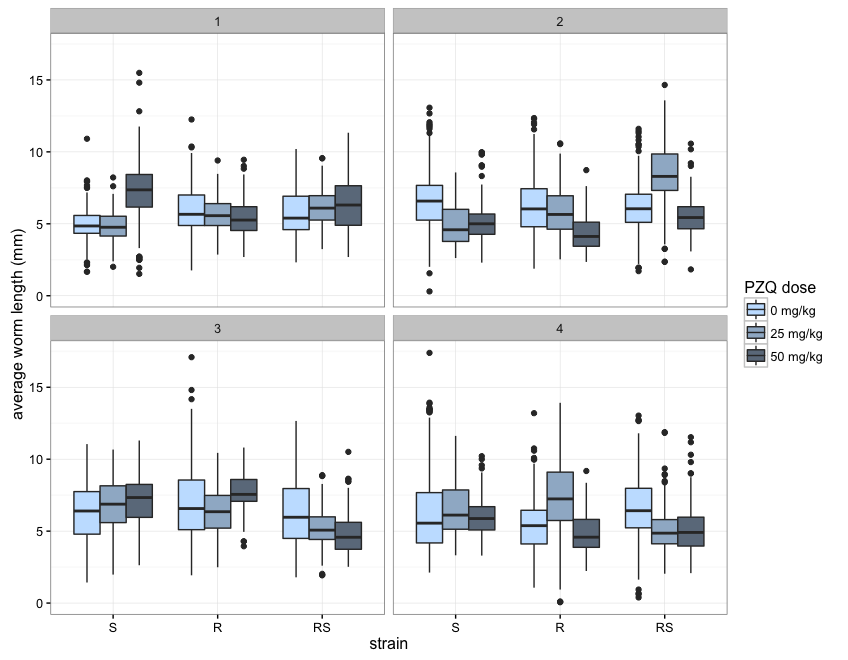
**

**Figure S2. Association between worm fecundity and total number of worms.** (A) Daily *Schistosoma mansoni* miracidia per worm compared with total number of worms and (B) Daily *S. mansoni* miracidia per worm pair compared with total number of worm pairs within each mouse. Daily miracidia production was not significantly linked to either total adult worms (A) or worm pairs (B).

**Figure S3. Association between virulence and the number of miracidia obtained.** Virulence (here presented as percentage of total body weight that was spleen and liver combined) is not directly linked total number of miracidia (presented on a log scale for easier viewing).


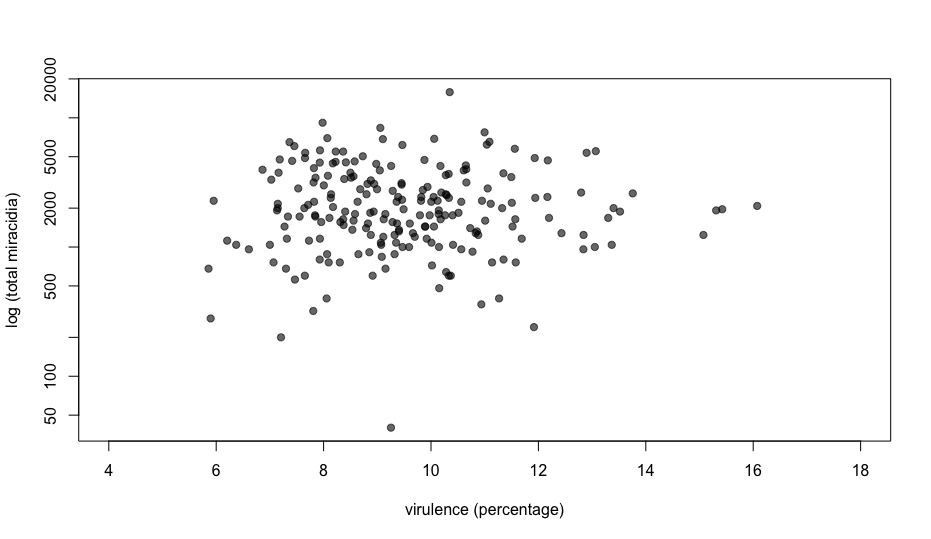

Supplement: Supplementary file 2 — Lamberton et al. 2017 IDoP Supplementary Figures 1, 2 and 3. (DOCX 322 kb) [file 40249_2017_324_MOESM2_ESM.docx]
